# COPD assessment test (CAT) scores as predictors of anxiety and depression in COPD patients

**DOI:** 10.6026/973206300221651

**Published:** 2026-03-31

**Authors:** Mahendra Singh Chouhan, Sourabh Jain, Satyendra Mishra, Sanjay Gupta, Talha Saad

**Affiliations:** 1 Department of Pediatrics, Bundelkhand Medical College, Sagar, Madhya Pradesh, India; 2 Department of Emergency Medicine, Bundelkhand Medical College, Sagar, Madhya Pradesh, India; 3 Department of Respiratory Medicine, Bundelkhand Medical College, Sagar, Madhya Pradesh, India; 4 Department of TB and Respiratory Diseases, Chief Medical Officer (Senior Administrative Grade), NITRD, Delhi, India

**Keywords:** Chronic Obstructive Pulmonary Disease (COPD), CAT score, anxiety, depression, mental health, GAD-7, PHQ-9

## Abstract

Anxiety and depression commonly co-occur in COPD patients, negatively impacting their quality of life and health outcomes. The COPD Assessment Test (CAT) evaluates COPD severity but its potential to predict mental health issues is underexplored. Therefore, it is of interest to investigate the correlation between CAT scores and anxiety and depression in 160 COPD patients. Significant correlations were found between higher CAT scores and increased levels of anxiety and depression. Thus, we show that CAT scores can be integrated into mental health screening, facilitating early detection and management of comorbidities.

## Background:

Chronic Obstructive Pulmonary Disease (COPD) is a significant public health issue, contributing to high rates of morbidity and mortality globally [1]. Patients with COPD frequently suffer from comorbid anxiety and depression, which adversely affect their quality of life and complicate disease management [2]. The COPD Assessment Test (CAT) is a simple, patient-completed questionnaire that assesses the impact of COPD symptoms on health status [3]. Although primarily used to measure physical symptoms, the potential of the CAT score to predict mental health issues in COPD patients has not been fully explored. Psychological comorbidities such as anxiety and depression are common but often under-recognized in COPD patients, despite their significant impact on symptom burden, functional status, exacerbation frequency and health-related quality of life [4]. Routine mental health assessment in COPD is limited in busy clinical settings; therefore, simple integrated screening approaches are needed. As the COPD Assessment Test (CAT) reflects overall symptom burden and daily life impairment, it may serve as a practical tool to identify patients at higher risk of anxiety and depression during routine evaluation [5, 6]. Therefore, it is of interest to evaluate the relationship between Chronic Obstructive Pulmonary Disease Assessment Test (CAT) scores and the prevalence of anxiety and depression among patients with COPD.

## Methods:

## Study design and participants:

This cross-sectional study included 160 patients diagnosed with COPD. Participants were recruited from outpatient pulmonary clinics. Inclusion criteria were a confirmed diagnosis of COPD according to the Global Initiative for Chronic Obstructive Lung Disease (GOLD) criteria [1]. Exclusion criteria included other significant respiratory diseases, recent acute exacerbation of COPD, or cognitive impairment that would interfere with the completion of questionnaires. Ethical approval was obtained and informed consent was provided by all participants.

## Measures:

The CAT score was used to assess the severity of COPD symptoms [3]. Anxiety and depression were evaluated using the Generalized Anxiety Disorder-7 (GAD-7) and Patient Health Questionnaire-9 (PHQ-9) scales, respectively [4, 5]. The GAD-7 and PHQ-9 are validated tools commonly used to screen for anxiety and depression in clinical settings.

## Statistical analysis:

Data were analyzed using SPSS software. Descriptive statistics summarized the demographic and clinical characteristics of the participants. Pearson's correlation coefficient assessed the relationship between CAT scores and GAD-7 and PHQ-9 scores [6]. Chi-squared tests of independence evaluated the association between categorized CAT scores and the presence of anxiety and depression. A p-value < 0.05 was considered statistically significant.

## Results:

The data presented explores the associations between CAT scores, anxiety (measured by the GAD-7), and depression (measured by the PHQ-9), as well as the distribution of anxiety and depression severity across different CAT score categories. Table 1 (see PDF) shows the association between CAT scores and anxiety. Participants with lower CAT scores (<10) had a higher proportion of no anxiety (GAD-7: 0-4) and mild anxiety (GAD-7: 5-9). As CAT scores increased, there was a higher prevalence of moderate anxiety (GAD-7: 10-14). Specifically, 25% of participants with a CAT score between 20-30 experienced moderate anxiety, compared to 21% with CAT scores <10. Table 2 (see PDF) highlights the association between CAT scores and depression. Similar trends are seen, with lower CAT scores being associated with less depression. Participants with CAT scores <10 had the highest proportion of no depression (PHQ-9: 0-4). As CAT scores increased, the number of participants with mild to moderate depression (PHQ-9: 5-14) increased, especially for those with CAT scores between 20-30. Table 3 (see PDF) further explores the distribution of anxiety severity according to CAT score categories. A clear pattern emerges, with those in the lower CAT score categories (<10) having the highest proportion of no anxiety, while participants in higher CAT score categories (31-40) showed higher severity levels, including moderate and moderately severe anxiety. Table 4 (see PDF) shows the distribution of depression severity by CAT score categories. Similar to anxiety, depression severity increases as CAT scores rise. For CAT scores <10, most participants had no depression, while those with CAT scores between 21-30 had a larger proportion of participants experiencing mild to moderate depression. Table 5 (see PDF) summarizes the mean GAD-7 and PHQ-9 scores across CAT score categories. As expected, both GAD-7 and PHQ-9 scores increased with higher CAT scores. The mean GAD-7 score for those with CAT scores <10 was 4.1 ± 2.8, while for those with CAT scores between 31-40, it increased to 14.1 ± 6.0. Similarly, PHQ-9 scores increased from 4.6 ± 3.1 for CAT scores <10 to 15.4 ± 6.2 for CAT scores between 31-40. In summary, higher CAT scores are associated with more severe levels of anxiety and depression, as indicated by the GAD-7 and PHQ-9 scores. The findings suggest that CAT scores may serve as a useful predictor for the severity of anxiety and depression in individuals.

## Discussion:

The findings of the present study demonstrate a strong association between higher CAT scores and increased levels of anxiety and depression among COPD patients. This highlights that symptom burden, rather than respiratory impairment alone, plays a crucial role in the psychological well-being of individuals with COPD [7]. The significant positive correlations observed between CAT scores and both GAD-7 and PHQ-9 scores suggest that patients experiencing greater daily life impairment are more susceptible to anxiety and depressive symptoms. Similar observations have been reported in previous studies, where symptom severity and functional limitation were identified as key predictors of psychological distress in COPD patients [8]. The categorical analysis further revealed that patients with CAT scores ≥21 were more likely to exhibit moderate to moderately severe anxiety and depression. This graded increase across CAT categories supports the concept of a dose-response relationship between COPD symptom burden and mental health morbidity [9]. Such findings emphasize the clinical relevance of CAT score thresholds in identifying high-risk patients. Importantly, these results underscore the potential role of CAT as a dual-purpose screening tool in routine clinical practice. Since CAT is already widely used for COPD assessment, its integration with mental health evaluation may allow early identification of anxiety and depression without additional time or resource burden [6]. Early recognition and intervention are essential, as untreated psychological comorbidities are associated with poor treatment adherence, increased exacerbation frequency and reduced quality of life in COPD patients [10].

## Significance:

Integrating CAT scores with mental health assessments could enhance early detection of comorbid conditions in COPD patients.

## Limitations:

The cross-sectional design limits causal inference and the study's sample size may restrict generalizability.

## Conclusion:

We show that higher COPD Assessment Test (CAT) scores are significantly associated with increased anxiety and depression in COPD patients. CAT scores show a clear, graded relationship with the severity of psychological symptoms. Incorporating CAT scores into routine mental health screening may facilitate early identification and management of anxiety and depression, leading to improved comprehensive care and better clinical outcomes in patients with COPD.

## References

[1] https://goldcopd.org/2024-gold-report/.

[2] Siraj RA (2025). Medicina (Kaunas)..

[3] Sari CP (2020). J Pharm Bioallied Sci..

[4] Beech A, Singh D (2023). Int J Chron Obstruct Pulmon Dis..

[5] Al Wachami N (2024). Int J Chron Obstruct Pulmon Dis..

[6] Hermosa R (2025). Diagnostics..

[7] Di Marco F (2006). Respir Med..

[8] Van Manen JG (2002). Thorax..

[9] Ghobadi H (2012). Tanaffos..

[10] Blakemore A (2014). Int J Chron Obstruct Pulmon Dis..

